# On recursion

**DOI:** 10.3389/fpsyg.2013.01017

**Published:** 2014-01-08

**Authors:** Jeffrey Watumull, Marc D. Hauser, Ian G. Roberts, Norbert Hornstein

**Affiliations:** ^1^Department of Linguistics and Philosophy, Massachusetts Institute of TechnologyCambridge, MA, USA; ^2^Department of Theoretical and Applied Linguistics, University of CambridgeCambridge, UK; ^3^Risk-EraserWest Falmouth, MA, USA; ^4^Department of Linguistics, University of MarylandCollege Park, MD, USA

**Keywords:** computability, FLN/FLB, Gödel, induction, recursion, syntax, Turing

## Abstract

It is a truism that conceptual understanding of a hypothesis is required for its empirical investigation. However, the concept of recursion as articulated in the context of linguistic analysis has been perennially confused. Nowhere has this been more evident than in attempts to critique and extend Hauseretal's. (2002) articulation. These authors put forward the hypothesis that what is uniquely human and unique to the faculty of language—the faculty of language in the narrow sense (FLN)—is a recursive system that generates and maps syntactic objects to conceptual-intentional and sensory-motor systems. This thesis was based on the standard mathematical definition of recursion as understood by Gödel and Turing, and yet has commonly been interpreted in other ways, most notably and incorrectly as a thesis about the capacity for syntactic embedding. As we explain, the recursiveness of a function is defined independent of such output, whether infinite or finite, embedded or unembedded—existent or non-existent. And to the extent that embedding is a sufficient, though not necessary, diagnostic of recursion, it has not been established that the apparent restriction on embedding in some languages is of any theoretical import. Misunderstanding of these facts has generated research that is often irrelevant to the FLN thesis as well as to other theories of language competence that focus on its generative power of expression. This essay is an attempt to bring conceptual clarity to such discussions as well as to future empirical investigations by explaining three criterial properties of recursion: computability (i.e., rules in intension rather than lists in extension); definition by induction (i.e., rules strongly generative of structure); and mathematical induction (i.e., rules for the principled—and potentially unbounded—expansion of strongly generated structure). By these necessary and sufficient criteria, the grammars of all natural languages are recursive.

## Three properties of recursion

The theory of recursive functions was propounded in the foundations of mathematics. One of the profoundest of foundational problems—Hilbert's decision problem (*Entscheidungsproblem*)—was to formulate a procedure (*Entscheidungsverfahren*) that, in a finite number of steps, would decide the validity of a given logical expression. More generally, from a logical formalism of finitely specifiable axioms and theories, such a procedure would decide—i.e., *compute*, hence *explain*—potentially infinite non-arbitrary sets of theorems and data in systems of the formal and natural sciences. “Once [this] logical formalism is established one can expect that a systematic, so-to-say computational treatment of logical formulas is possible” (Hilbert and Ackermann, 1928: 72). It only remained to formalize the intuitive concept of *computability*.

### Computability

Turing (1936) demonstrated that computational treatments could be established for particular decision problems, but that no general decision procedure exists. Thus concluded the Hilbertian program. However, of greater significance than this negative result was that, in its application to the decision problem, Turing's mathematics had formalized the intuitive concept of a computation—equivalently, a *generative procedure*—in the form of his *automatic machine* (now called a *Turing machine*): a mathematical object—an abstract computer—represented functionally by a *control unit* (a finite program of rules, states, and symbols), a *tape* (an unbounded memory), and a *read/write head* (a mechanism for decoding, encoding, and manipulating symbols on the tape). In a stepwise process analogous to proof construction, the machine deterministically generates outputs (analogous to theorems) given inputs (which, with initial conditions, form a set analogous to axioms) by returning—*recursing*—intermediate results (analogous to lines or lemmas) according to its programmed rules.

It is of fundamental importance to understand that the non-arbitrary set generated by a recursive function qua Turing machine need not be represented as an output; to recursively *generate* a set is not to *produce* it. The function is defined in *intension*: it specifies the conditions some object would need to satisfy to be subsumed in the set, and it is possible that no such objects are presented. This notion of intension is realized in a Turing machine as rules for *conditional branching*: e.g., “IF in state *q*_*i*_, reading symbol *x*_*i*_ on the tape, THEN write *y*_*i*_, move one space, transition to state *q*_*j*_.” The machine produces a set of outputs if and only if it enters the defined configurations, but that set—the set of possible outputs—is *determined* (generated) by the rules “in advance” of—indeed independent of—any input. For example, independent of its potential application to particular inputs, a rule of arithmetic determines (generates) a range of numbers (i.e., the non-arbitrary set of possible outputs) given a domain of inputs.

It is because of the aforementioned insights from Turing that we define language as *I-language*: a function in *intension*, *internal* to the mind/brain of an *individual* of the species *Homo sapiens sapiens.* I-language is to be distinguished from *E-language*: one way to think of E-language, departing from the terminology of Chomsky (1986), is as the function defined in *extension*. The extension of I-language can be defined as the set of objects it generates and thereby constrains (i.e., the set of outputs it could in principle produce, including any it actually does). We can further illustrate the I- v. E- distinction in the case of language by drawing an analogy to arithmetic. We can define *I-arithmetic*—represented internal to the mind/brain of an individual of the species *Homo sapiens sapiens*—as the function in intension that generates as its extension a set of arithmetical theorems (*E-arithmetic*). The latter may not be represented anywhere (internally or externally); it is non-etheless the set generated by the former in the sense that the extension is deterministically specified by the intension. If I-arithmetic were encoded as a computer program (a finite amount of information), the standard ontological assumption in computer science and information theory would be that E-arithmetic—which would require an infinite number of bits to fully enumerate—is *compressed* into the program. If I-arithmetic were to output a set, the members would be those and only those that satisfy the specified conditions (i.e., the intension) of the generative function; in this way E-arithmetic is constrained. Identical logic defines I-language into which E-language is compressed: the generative function—a finite conditional branching program—determines a (potentially infinite) set of structured expressions that could but need not be enumerated^1^. Thus, the I-language/E-language distinction is fundamental to understanding the nature of FLN (as a function in intension) and its uniqueness (i.e., as far as we know, no such distinction applies to the claimed analogues/homologues of language in non-human animals).

The range of a recursive function can be infinite, as with the arithmetical rule. The computation can run to infinity, but its rules are finitely specified and at any step in the computation only a finite amount of tape has been processed. The finiteness of the machine is fundamental: the set of theorems or data derivable or predictable in a consistent system or theory is non-arbitrary such that an extensional definition (a list of the theorems/data) cannot suffice; a list does not derive and thereby delimit (and thereby explain) the set. Therefore, it is necessary to define the set intensionally by a finite procedure (a rule) to derive or predict what things satisfy the conditions to be subsumed in the set or equivalently to generate (describe) all and only those things the set subsumes. In other words, if a set is non-arbitrary, then there must exist a reason why it subsumes all and only those things it does; and the rule is the reason.

Computable functions are therefore those “calculable by finite means” (Turing, 1936: 230), specifically by a Turing machine. With this elegantly elementary model of computation, the general concept of a *formal system* was established. Such systems are generative, and thus explicative of sets of theorems/data. These systems therefore constitute the ontological and epistemological foundations of the formal and natural sciences.

### Induction

Turing proceeded to prove the mathematical equivalence of computability to the *effective-calculability* (*λ-definability*) which Church (1936) had demonstrated to be mathematically equivalent to the *recursiveness* of Gödel (1934/1986). Gödel had embroidered the primitive notion of recursion whereby “[a] number theoretic function ϕ is said to be *recursive* if there is a finite sequence of number-theoretic functions ϕ_1_, ϕ_2_, …, ϕ_*n*_ that ends with ϕ and has the property that every function ϕ_*n*_ of the sequence is recursively defined in terms of […] preceding functions, or […] is the successor function *x*+1” (Gödel, 1931/1986: 159). We next explain the significance of this rather dense definition for our discussion.

Gödel's first property of a recursive function—its specification in a finite sequence—is that of Turing computability. The second and third properties—the function is defined in terms of preceding functions or reduces to the successor function—are those of induction, in its two senses: *definition by induction* and *mathematical induction*; the distinction between these related concepts can be seen in the specific recursive system of generative grammar.

The original formulation of a generative grammar was not as a Turing machine but as the formally equivalent *rewrite rules* of Post (1947). Rewrite rules of the form ϕ → ψ (“rewrite ϕ as ψ”) determine successive tape-machine configurations to derive syntactic structures and thus, as with Turing machines, function analogously to the rules of a proof. The derivation of a syntactic structure can be defined as the “running through” (Chomsky, 1955: 67) of rewrite rules [n.b., *recursion* is from the Latin *recursio* (“running back”)]: “A derivation is thus roughly analogous to a proof with Σ,” a finite set of initial symbols, “taken as the axiom system and F,” the finite set of rewrite rules, “[taken] as the rules of inference” (Chomsky, 1956: 117).

The Post formalism represents explicitly the recursiveness of a generative grammar, with outputs recursed (returned) as inputs in the form of recursion applied by Gödel and represented in the stepwise computation of a Turing machine: i.e., definition by induction (*definition by recursion*) whereby a function *f* is defined for an argument *x* by a previously defined value (e.g., *f*(*y*), *y* < x) so as to *strongly generate* increasingly complex structures carried forward on the tape. The strong generation of structures studied in formal linguistics is contradistinguished from the *weak generation* of strings studied in formal language theory; and only strong generation is of relevance to research on natural language [see Chomsky (2007) on the inapplicability of formal language theory to natural language]. In other words, a grammar strongly generates hierarchically structured expressions and weakly generates the corresponding strings. It is a structure, not a string, that represents grammatical information. This information can, in turn, be mapped via formal semantics and morphology-phonology to the conceptual-intentional and sensory-motor systems. This mapping is supported by the fact that one string can correspond to many structures (in a many-one function). Consider the string *the boy saw the man with binoculars*. This string is two-ways ambiguous because it corresponds to two possible structures representing two possible interpretations: (i) {{the, boy}, {saw, {the, {man, {with, binoculars}}}}}; (ii) {{the, boy}, {{saw, {the, man}}, {with, binoculars}}}, Thus, work on artificial language learning that is based on formal language theory, with its restriction to weak generation (strings), makes it largely irrelevant to the strong generation (structures) of all natural languages. In this sense, and contrary to Fitch and Friederici (2012), we do not see too many applications of formal language theory to have been all that profitable in clarifying concepts, formulating and testing hypotheses, or opening new frontiers of research. However, there are important exceptions (e.g., Berwick, 1984; Stabler, 2010).

Definition by induction is related to the form of recursion as mathematical induction from bounded to unbounded: “by a *grammar* we mean a set of rules that (in particular) recursively specify the sentences of a language. In general, each of the rules we need will be of the form ϕ_1_, …, ϕ_*n*_ → ϕ_*n* + 1_, […] where each of the ϕ_*i*_ is a structure […] and the relation → is to be interpreted as expressing the fact that if our process of recursive specification generates the structures ϕ_1_, …, ϕ_*n*_ then it also generates the structure ϕ_*n* + 1_” (Chomsky and Miller, 1963: 284). This formulation is analogous to the successor function that, like other similar functions, has the potential to generate an unbounded output. Syntactic structures can be infinitely expanded by, among many other means, conjunctions of phrases/clauses, adjectival modifiers, prepositional modifiers, the mergers of relative and complement clauses, and combinations of these rules [see Jackendoff (2003) for examples]. We turn next to a discussion of bounded and unbounded outputs, and their relationship to the underlying generative function.

### (Un)boundedness

The mathematical induction from bounded to unbounded is perhaps the most misunderstood aspect of a recursive procedure. Three facts are critical to proper understanding.

First, a recursive function may *generate* an infinite set yet only *produce* a finite output because of arbitrary constraints. For instance, Turing formulated a machine (a recursive function) for the effective calculability of π. The decimal expansion of π is infinite (i.e., 3.14…) such that the machine requires infinite steps and infinite memory to generate its value as would a machine for generating an infinite set of syntactic structures. Nevertheless, the Turing machine generates or determines that the third digit in the decimal expansion of π is 1 even if its tape can only represent the first two digits; technically, even if the machine is tapeless, it generates the third (and fourth and fifth…) digit (n.b., encoded with conditional branching, the machine would write the digits if provided with sufficient tape). Analogously, the grammar of a language L generates L in its infinity—i.e., I-language generates E-language—regardless of the fact that only a finite subset of its structures can ever be physically produced. For instance, there exist infinitely many grammatical expansions of a 1300-word monster sentence from Faulkner's *Absalom, Absalom!*, though most will never be produced [see Pinker (1999)]. It is thus a fundamental fallacy to conclude that “the use of infinity as a tool for proofs in mathematics […] leaves such proofs technically irrelevant in the real world of finite brains and finite time” (Fitch and Friederici, 2012: 1933): *finite brains running in finite time literally do generate infinite sets*. Equally arbitrary to physical limitations are formal stipulations: e.g., capping the calculation and/or representation of a decimal expansion to two digits; e.g., capping syntactic embedding to some finite depth.

Second, because the range of a recursive function is by definition non-arbitrary, any arbitrarily limited output can be expanded in a principled manner. So “even though we have a finite brain, that brain is really more like the control unit for […] a Turing machine in the sense that although we have a finite control unit for a brain, nevertheless we can use indefinite amounts of memory that are given to us externally,” e.g., as a tape, “to perform more and more complex computations[…]. We do not have to learn anything new to extend our capacities in this way” (Chomsky, 2004: 41–42). We do not have to learn anything new because our computations are “*completely determined*” by programs enabling conditional branching, i.e., “a note of instructions […] explaining how the work is to be continued” given any possible input, which may never be given; hence *conditional* branching. “This note is the counterpart of the ‘state of mind’ [and] determines the possible behavior of the machine” (Turing, 1936: 253, 231). Such programs are explicitly rejected in connectionism (e.g., Elman, 1991; Siegelmann, 2013) and apparently absent in non-human animal cognition (e.g., Rey et al., 2012; Watumull et al., 2013). Whether these uniquely human programs emerged specific to language and were thereafter exapted to other domains (e.g., mathematics, music, morality, etc.) or were from the beginning domain general (e.g., Corballis, 2011) is an interesting question [see DeWitt (2013)], but one that is outside of the present paper's aims. Whatever their origin, these recursive programs provided our capacity for thought and expression with a uniquely powerful upgrade.

Third, the representation (extension) of the set generated is immaterial to the form (intension) of its generation. For instance, given that recursiveness is a property of the procedure applicable to any input rather than a property of potential output, equating recursion with syntactic embedding is simply a fallacy.

### Recapitulation

The core computational mechanisms of recursion, proposed to be constitutive of FLN, are: (i) computability, (ii) definition by induction, and (iii) mathematical induction. A computable function is necessary to derive sets; a list is epistemologically and, more importantly, ontologically meaningless. That the function is defined inductively enables it to strongly generate increasingly structured expressions. And with mathematical induction, the computable generation of such expressions is unbounded.

## Recursion in human and non-human animals

Given the explication of recursion in the previous section, we turn next to two lines of research that have been headlined as knockouts of the FLN thesis, as well as earlier formulations of *Universal Grammar* (UG) (the theory of the genetic endowment for language). In particular, we discuss why claims of languages lacking recursive expressions, though of interest, say nothing about recursive functions because output systems are simply not relevant to the instantiation of this capacity in all human brains. Conversely, we discuss why claims that animals are capable of processing embedded expressions, though interesting, do not take down the uniqueness claim that underpins the FLN thesis. Embedding, though of interest as a computational capacity, is not synonymous with recursion, and nor is embedding part of the original FLN thesis.

### Caps and gaps

Consider a recent critique of FLN-style theories: “there is little evidence that unlimited recursion, understood as center-embedding, is typical of natural language syntax. [T]his fits ill with the claim (Hauser et al., 2002) […] that ‘recursion’ (understood as embedding) may be the one crucial domain-specific feature of linguistic ability” (Levinson, 2013: 149, 152). This quote embodies the conceptual confusions discussed in the earlier sections above.

First, as discussed in section Three Properties of Recursion, to understand recursion as embedding is actually to misunderstand recursion: to equate recursion—a property of the generative procedure (applicable to any input)—with possible properties (e.g., embedded structure) of its (potential) output is simply a mathematical error. But even accepting properties of the output as indicative of a recursive mechanism, embedding is not dispositive, as linguistic typologists have long known: e.g., “In a large number of Australian languages, the principal responsibility for productive recursion in syntax is shouldered by [an] adjoined relative clause. It is typically marked as subordinate in some way, but its surface position with respect to the main clause is marginal rather than embedded” (Hale, 1976: 78); n.b., here Hale is (correctly) identifying the mathematical inductive aspect of recursion.

Second, any limitations on depth of embedding in structures that FLN does generate can only be arbitrary—i.e., arbitrary with respect to the generative function—given that recursiveness—i.e., computability, definition by induction, and mathematical induction—is an independent property of the function. For instance, it would be an (interesting but) arbitrary fact of the morphology of Kayardild if its oblique case marker “blocks recursion at one level deep” (Levinson, 2013: 152); embedding—not to be conflated with recursion—would be “capped at a very shallow level” (Levinson, 2013: 157). It would be analogously arbitrary if in some arithmetical system it were impossible to represent decimal expansions of length *n*, *n* > 2, such that π could only ever be expanded to 3.14. The mathematical fact would remain that a computable function for π generates its infinite decimal expansion (n.b., this fact distinguishes a computable function from a lookup table listing the decimal expansion to some finite length); unboundedness is a property of generative competence, not its application in performance.

Third, it is false that “boundedness is principled” if for instance it is possible for the generative function only to “produc[e] a maximum phrase consisting of the verb's lexical frame plus as much as one modifier word per constituent of the phrase and up to one prepositional adjunct phrase” (Everett, 2012: 558); incidentally, the bound is claimed only for “sentential syntax,” but of course syntax—and recursion—extends “super-sententially” (as we will discuss). This function is demonstrably computable: i.e., the set of possible phrases is non-arbitrary and, even if finite, contains too many members to be listed as a lookup table; thus it must be generated by a finitary (recursive) procedure. The function is defined by induction: i.e., outputs are recursed (carried forward on tape) as inputs to strongly generate structured expressions; thus the process is not a form of *iteration* (equivalently *tail recursion*) as claimed^2^. And finally, the function is mathematically inductive: i.e., unboundedness would emerge with relaxation of the arbitrary lexical restrictions; furthermore, even with such restrictions, it has not been demonstrated that the number of arguments per verb and the number of modifiable constituents is bounded by principle. In short, this function is recursive.

Ultimately, any boundedness is demonstrably arbitrary as proved by the undisputed fact that recursion is unbounded in some (i.e., most or, as we submit, all) languages: i.e., it follows from mathematical law that recursion is unlearnable and thus must be part of the species endowment (UG), and thus universal^3^. The number faculty is analogous:

“[Counting] is universal, and whether or not a conventionalized inventory of numerals exists in a given language depends upon the extent to which exact enumeration is of practical use or necessity to the people who speak the language. One might look upon the Walbiri lack of conventionalized numerals as a gap in the inventory of cultural items—since the principle which underlies counting is present,” i.e., genetically determined, “filling the gap is a rather trivial matter. [C]ertain cultural items can be said to be universal even though they may not be included in the inventory of cultural items for particular communities. This is not a contradiction if one bears in mind that what is universal is the concept, not some conventionalized manifestation of it” (Hale, 1975: 296).

Therefore even if it were true that “[t]he upper limit of a Pirahã sentence is a lexical frame with modifiers [a]nd up to two […] additional sentence-level or verb-level prepositional adjuncts” (Everett, 2012: 560), nothing would follow for the universality of recursion. And incidentally, to reiterate, it is undisputed that all languages are recursively unbounded at the super-sentential (discourse) level; and the sentential/super-sentential distinction is artificial, as we discuss in section Universality.

### Evolution

The FLN thesis is a proposal about what is unique to our species and unique to the faculty of language. As in any claim about uniqueness, comparative research is required—in this case, comparing the capacities of different species as well as different domains of knowledge other than language within our own species. To suggest that only humans are endowed with recursive capacities that map to the sensory-motor and conceptual-intentional systems is to formulate a challenge for comparative work, a challenge that requires critical observations and experiments. To this end, Fitch and Hauser (2004) sought to develop a research program that would potentially enable a test of this hypothesis. Due to its computational rigor and clarity, they started with the Chomsky hierarchy (Chomsky, 1959a). More specifically, they designed an experiment based on two features that would facilitate comparison with humans, both young and old: (1) an experimental procedure that required no training and thus could elicit evidence of spontaneous processing, akin to child language acquisition; (2) stimuli designed to distinguish between different levels of computational power, specifically, one pattern mapping onto a finite-state grammar as opposed to one mapping onto a phrase-structure grammar. This was a test of computational power, not the three criterial properties of recursion; establishing the former is necessary to exploring the latter. Based on both the introduction to this paper, and its conclusions, independently of the particular results, not even the most positive evidence would serve to knockout the FLN thesis. Rather, this experiment was designed to provide a different approach to the problem of comparing across species, one that might enable a more direct comparison between humans and non-human animals. Results showed that cotton-top tamarins spontaneously processed the finite-state grammar (i.e., AB^*n*^), but failed to process the phrase-structure grammar (i.e., A^*n*^B^*n*^). Fitch and Hauser concluded that the generative capacity required to fully process a phrase-structure may have been the bottleneck that we alone broke through.

Based on both the design and results of Fitch and Hauser's experiment with cotton-top tamarins, other studies on other species soon emerged (e.g., Gentner et al., 2006; Rey et al., 2012), using both similarly designed stimuli (i.e., “grammars”), but typically differently designed methodologies. Unfortunately, in our opinion, most of these studies misinterpreted the reasoning behind the Fitch and Hauser experiments, both in terms of methodological design as well as theoretical implications. Specifically, the majority of studies that followed used massive training, as opposed to spontaneous methods. Though these methods can show what an animal can do under these conditions (thousands of trials with reinforcement), these are not the conditions in which human children acquire language. No child forms a fully functional grammar based on exposure to 50,000 exemplars from an extremely narrow set of inputs, and then differentially reinforced for correct responses. This was an idea long ago put forward by Skinner (1957), and soundly taken apart by Chomsky (1959b). This is not to say, however, that the spontaneous looking time method is without serious flaws: e.g., the method only enables a small difference in conditions that may be biologically insignificant (i.e., differences of 1–2 s in looking time between conditions can give a statistically significant result); because studies of animals use small populations, it is not possible to test the variety of alternative explanations. Thus, the results using either method gain little traction in terms of meaningful comparison with human ontogeny.

In addition to the methodological departures, most of the studies using the Chomsky hierarchy as in Fitch and Hauser (2004) have falsely concluded that evidence of successful processing of embedding constitutes evidence against the FLN hypothesis. But as noted above, this conclusion simply does not follow: recursion and embedding are not synonymous; nor are generation and processing. Furthermore, all of the published work on embedding, even in the best cases, shows nothing like the structured expressions that FLN strongly generates.

So what would studies of non-human animals need to demonstrate to challenge the FLN hypothesis? Given the definition of recursion discussed in the previous sections, it would be necessary to show the *spontaneous* display of (i) computability, (ii) definition by induction, and (iii) mathematical induction. (i) Computability requires proof of a procedure: a conditional branching program that generates new and complex representations by combining and manipulating symbols, as in human language; this *productive* process is to be contrasted with the *retrieval* of representations from a lookup table (finite and innately specified or memorized), as in the calls of non-human primates. (ii) The computable function must be defined by induction: outputs must be carried forward and returned as inputs. For instance, animal navigation by path integration (dead reckoning) requires the carrying forward of vector values: displacements are summed to plot a path. However, to demonstrate equivalence with linguistic recursion, this process would need its outputs to be not only returned as inputs but also represented hierarchically. Summing vectors just generates another vector (in a tail recursive process), not a hierarchical structure over which can be defined complex relations (e.g., syntactic, semantic, phonological, etc.); this also implies discreteness of representations. (iii) Mathematical induction is seen in the jump from finite to infinite. This can be demonstrated by generalization beyond the exposure material (e.g., counting indefinitely beyond the training set) and by revealing an unbounded competence underlying bounded performance (e.g., human performance in processing syntactic structures increases indefinitely—with no changes to the internal program of competence—if time and access to external memory are increased, and so too should that of any non-human animal endowed with a comparable competence). Thus, the potential for productive future research is vast, but will rely on the creation of far more clever methods than presently available.

## Universality

If recursion is as fundamental to language as we have argued, some would say it could be “discounted” as a linguistic universal, for it would be one of those “features of language that are universal by definition—that is, we would not call the object in question a *language* if it lacked these properties [and others] that all languages need in order to be adequately expressive instruments” (Evans and Levinson, 2009: 437). But this argument is unsound.

First, it is not as if a language precedes its properties, specifying those it requires to be “adequately expressive,” thereby satisfying some Lamarckian “felt need.” FLN formed by the emergence and organization of properties into a system that may or may not be adequate for any expression.

Second, and most importantly, given the existence of such systems, the non-trivial task is to discover those properties *definitional* of these systems: i.e., the system must be defined in intension. As explicated by Carnap(1955: 42), “the intension of a predicate ‘Q’ […] is the general condition which an object y must fulfill in order for [the ascription of] ‘Q’ to y.” What are the conditions an object must satisfy for it to be classed as a “language”? These conditions (satisfiable by particular properties) are the desiderata of the language sciences—indeed intensional definitions are the desiderata of science generally (e.g., “What is *life*?”). In other words, it would be odd to dismiss from consideration “features of language that are universal by definition” because the definition of language is an empirical discovery: the properties of the definition are thus *architectural universals*—the profoundest of all. Recursion, we submit, is an architectural universal.

### Super-sentential syntax

Interestingly, it is universally recognized that recursion is an architectural universal (from Hauser et al., 2002 to Everett, 2012 and Levinson, 2013). The only issue is whether the universality of recursion is exclusively super-sentential (e.g., recursive discourse structure)^4^. In our judgment this is a non-issue: there is no difference in kind between “sentential” and “super-sentential” recursion. A proper defense of this judgment would exceed the bounds of this essay, so here we merely adumbrate our argument.

In current generative grammar, the “sentence” exists only informally as a description of an intuitively perceived “unit” of syntactic computation. But technically there is posited to exist only the irreducibly elementary *f*_merge_ function (central to FLN) and the sets of structured expressions of various sizes it generates. (Any adequate theory of language must posit some recursive mechanism. Perhaps it need not be *f*_merge_, but it must be propertied with the three forms of recursion that are represented in *f*_merge_: computability, definition by induction, and mathematical induction.) *f*_merge_ is a function “calculable by finite means,” i.e., a computable function: *f*_merge_(X, Y) = {X, Y}; given syntactic objects X, Y, *f*_merge_ forms the set {X, Y}. {X, Y} can be recursed to merge with a syntactic object Z to strongly generate {Z, {X, Y}}. {Z, {X, Y}} is thus defined recursively (i.e., by induction) in a stepwise strongly generative process creating increasing complexity^5^. And obviously a quintessential property of linguistic recursion—as it appears in FLN—is the induction from bounded to unbounded: *f*_merge_(W, {Z, {X, Y}}) = {W, {Z, {X, Y}}}; *f*_merge_(T, {W, {Z, {X, Y}}}) = {T, {W, {Z, {X, Y}}}}; …. This requires the logical architecture of a Turing machine with its tape to carry forward intermediate outputs to be recursed as inputs; and *f*_merge_ is naturally modeled as the “write” function of the machine.

In purely syntactic (formal) structures, e.g., {Z, {X, Y}}, we see no reason—nor has any been given—for stipulating that “sentences” cannot be substituted for the variables, thereby generating a “discourse” structure (informally, a “paragraph”). Such a structure, however it is represented^6^ must of course be *generated* (not fetched from a lookup table). The function generative of this structure either is *f*_merge_ or it is not *f*_merge_. Clearly, unless we have truly compelling evidence that it is not *f*_merge_, we should assume on general grounds of parsimony that it is *f*_merge_. We are unaware of any evidence that the function in question is not *f*_merge_, so we should assume that it is *f*_merge_. We therefore assume that discourse structures are recursively generated, but much remains to be fleshed out [see Watumull and Roberts (under review) for a fuller treatment of super-sentential syntax].

## Conclusion

Support for rejection of the FLN thesis speaks directly to our conception of human nature (i.e., those traits unique to and definitional of the species) and the nature of the universe (i.e., the ontological interconnection of mathematics and biology). Understanding this thesis is thus of fundamental importance. And such an understanding is possible only if one of its core concepts—recursion—is precisified formally into its three criterial properties as discovered and expounded by Turing and Gödel: the computability of rules generative of non-arbitrary sets; definition by induction enabling the strong generation of increasingly structured expressions; and mathematical induction for the principled (and potentially unbounded) expansion of the generated sets of structures. So understood, recursion is the foundational linguistic universal.

### Conflict of interest statement

The authors declare that the research was conducted in the absence of any commercial or financial relationships that could be construed as a potential conflict of interest.

## Footnotes

^1^Given that recursive procedures define linguistic and mathematical cognition, investigations into the degree of neural overlap of these domains could bear upon the FLN hypothesis.

^2^A tail recursive procedure applies only to the end (the “tail”) of the immediately preceding output; the entire “derivational history” is not carried forward on the Turing tape. The strong generation of Pirahã sentences thus goes beyond tail recursion. (Note that tail recursion is still recursion, so no claim remains that recursion is absent from Pirahã.)

^3^We assume with the majority of researchers that humans do not vary genetically in their linguistic capacities. But even if there were variation, recursion in some form or another would need to be encoded genetically because it cannot be learned even in principle. If the capacity for recursion—computability *and* inductive definitions *and* mathematical induction—were knocked out of the genome in toto, the phenotypic effects would be catastrophic: language would crash and the systems it enables would collapse; ultimately, any cognitive domain with computable and/or inductive properties would fail.

^4^For us a *discourse* structure can be informally described as a “paragraph,” not a “dialog” as analyzed in Levinson (2013) and criticized in Legate et al. (2013); that is, we define a discourse to be a combination of “sentences” internal to one language user rather than an interaction between language users.

^5^As an exercise, substitute *the* for X, *apple* for Y, and *eat* for Z to generate a verb phrase; this recursively generated structured expression could be informally described as “sub-sentential.”

^6^A flat structure generated by an *n*-ary function could assume the form {{To each his tragic own}, {Socrates drank the hemlock}, {I ate the apple}}. More probable is a hierarchical structure generated by a binary function of the form {{To each his tragic own}, {{Socrates drank the hemlock}, {I ate the apple}}}. Thus, we assume that a discourse structure would be binary-branching. Most probable and interesting is a hierarchical structure generated by a binary function via null (i.e., unpronounced) coordinators (“&”): {{To each his tragic own}, {&, {{Socrates drank the hemlock}, {&, {I ate the apple}}}}}. If the structure is formed by such conjunctions, then no sentence-internal constituent could c-command out of its sentence into another one, consistent with the evidence.
